# Enhancing Public Health Capacity by using Epidemiological Teams in a Public Health Setting

**DOI:** 10.7759/cureus.1381

**Published:** 2017-06-22

**Authors:** Hammad Akram

**Affiliations:** 1 Public Health, Independent Researcher

**Keywords:** epidemiology, public health, epidemiological teams, epi teams, response, public health response, outbreak, disaster, enhanced surveillance, capabilities

## Abstract

Epidemiological (Epi) team is a team of diverse and multidisciplinary public health professionals who provide support and strength to the capacity of an agency during an event where the need for resources is beyond the routine capability. The Epi teams consist of a staff with skills ranging from specialty areas such as epidemiology, environmental health, health preparedness, nursing, health education, laboratory technology, etc. Establishment of Epi teams could be a very useful addition to a public health department and could lead to the early, efficient, and effective response to public health emergencies.

## Editorial

In a public health setting, epidemiology is considered to be an important specialty that helps to understand the distribution and determinants of health-related events and outcomes [1]. Field epidemiological operations are a critical part of a public health program as they could lead to the timely collection of public health data, identification of an outbreak, assessment of resources and their appropriate allocation, and effectively implementing control measures.

Most of the public health departments in the Western world have sufficient resources and staff to monitor epidemiological activities in their jurisdictions; however, in alternate situations (insufficient resources or lack of staff), an epidemiological (Epi) team could be utilized to achieve public health and epidemiological goals. An Epi team is a team of diverse and multidisciplinary public health professionals who provide support and strength to the capacity of an agency during an event where the need for resources is beyond the routine capability. In most scenarios, the Epi teams could be useful to implement an enhanced outbreak response plan [2]. The plan includes initial or baseline surveillance which could lead to the identification of an outbreak, post-identification enhanced surveillance to capture additional cases, investigative activities to identify sources and exposed contacts, executing control measures, and utilizing surveillance data to assess the situation. Furthermore, the teams can also assist in routine surveillance and review of the statistics of the existing disease. In addition to the communicable disease outbreaks, the teams can be deployed as a part of an epidemiological response during a natural or man-made disaster, preparedness planning, and enhanced response activities for diverse public health threats.

The Epi team is usually led by an epidemiologist or a professional experienced in outbreak investigation and public health epidemiology. The Epi team consists of clinical/nursing staff, environmental health or sanitation specialists, health educators, preparedness staff, planners, a public information officer, and Epi-trained staff (public health and/or administrative staff trained in epidemiological principles). The team is usually led by an epidemiologist or designee who directly coordinates with the program manager, director, or appropriate leadership of an agency to facilitate the incident-related activities. The teams can be categorized according to their expertise and academic background. For e.g., a nursing team can assist in the clinical aspects of the situation such as collecting data through clinical history, collecting client samples, recommending infection control measures, educating clients, assisting in vaccination, etc. Environmental health professionals can provide their expertise in the identification of an environmental agent (infectious or toxins), the collection of samples through field investigation, and implementation of control measures accordingly. The Epi-trained staff can perform data collection and entry, and assist in maintaining the line listing of cases and controls. The public information officer plays an important role in handling all media inquiries and other communication activities. All teams or involved staff members are also responsible for providing the regular situational briefing to the incident leader (epidemiologist and/or designee).

The role of the epidemiologist is to lead the team during a response, hold regular training and/or meetings throughout the year, and establish internal and external stakeholder relationships to facilitate the surveillance of epidemiological activities. All Epi team staff should be trained in the incident command system (ICS) to understand how to manage projects or events efficiently and effectively [3].Furthermore, the teams should also be trained on the Health Insurance Portability and Accountability Act (HIPAA) to learn basic privacy principles and security protection of health information [4].

In summary, Epi teams are responsible for disease surveillance, detection, investigation, control, and education. Establishment of Epi teams could be a very useful addition to a public health department. Increasing the capacity and resources of a small or local health department could lead to the early, efficient, and effective response to public health emergencies.
